# Extracellular vesicle-encapsulated miR-22-3p from bone marrow mesenchymal stem cell promotes osteogenic differentiation via FTO inhibition

**DOI:** 10.1186/s13287-020-01707-6

**Published:** 2020-06-10

**Authors:** Xueliang Zhang, Yongping Wang, Haiyan Zhao, Xingwen Han, Tong Zhao, Peng Qu, Guangjie Li, Wenji Wang

**Affiliations:** Department of Orthopedics, The First Hospital of Lanzhou University, No. 1, Donggang West Road, Lanzhou, 730000 Gansu Province People’s Republic of China

**Keywords:** Bone marrow mesenchymal stem cells, Extracellular vesicles, miR-22-3p, FTO, MYC, Osteogenic differentiation, PI3K/AKT pathway, Osteoporosis mouse model

## Abstract

**Background:**

Bone marrow mesenchymal stem cells (BMSCs) exhibit the capacity to self-renew and differentiate into multi-lineage cell types, including osteoblasts, which are crucial regulators of fracture healing. Thus, this study aims to investigate the effect of microRNA (miR)-22-3p from BMSC-derived EVs on osteogenic differentiation and its underlying mechanism.

**Methods:**

Extracellular vesicles (EVs) were isolated from BMSCs and taken up with BMSCs. Dual-luciferase reporter gene assay was used to verify the binding relationship between miR-22-3p and FTO. Loss- and gain-of-function experiments were performed to determine the roles of EV-delivered miR-22-3p and FTO in osteogenic differentiation as well as their regulatory role in the MYC/PI3K/AKT axis. To determine the osteogenic differentiation, ALP and ARS stainings were conducted, and the levels of RUNX2, OCN, and OPN level were determined. In vivo experiment was conducted to determine the function of EV-delivered miR-22-3p and FTO in osteogenic differentiation, followed by ALP and ARS staining.

**Results:**

miR-22-3p expression was repressed, while FTO expression was elevated in the ovariectomized mouse model. Overexpression of miR-22-3p, EV-delivered miR-22-3p, increased ALP activity and matrix mineralization of BMSCs and promoted RUNX2, OCN, and OPN expressions in BMSCs. miR-22-3p negatively targeted FTO expression. FTO silencing rescued the suppressed osteogenic differentiation by EV-delivered miR-22-3p inhibitor. FTO repression inactivated the MYC/PI3K/AKT pathway, thereby enhancing osteogenic differentiation both in vivo and in vitro.

**Conclusion:**

In summary, miR-22-3p delivered by BMSC-derived EVs could result in the inhibition of the MYC/PI3K/AKT pathway, thereby promoting osteogenic differentiation via FTO repression.

## Background

Bone marrow mesenchymal stem cells (BMSCs) belong to a population of self-renewing multipotent cells and can differentiate along several lineages like osteogenic lineage in response to stimulation by multiple environmental factors and play a critical role in tissue regeneration [1]. Because of their osteogenic potential, BMSCs are able to undergo self-renewal and differentiate into tissues of mesenchymal origin, making them significant contributors during bone repair [2, 3]. Moreover, it was reported that BMSC osteogenesis promotes calvarial bone regeneration [4]. Based on the findings from a prior study, BMSC-derived extracellular vesicles (EVs) assist osteogenic differentiation in steroid-induced femoral head necrosis [5].

EVs are nanoscale (40–150 nm) membranate vesicles that are widely distributed across the body and have been identified as extracellular signal transduction molecules following extensive research [6]. Proteins, lipids, mRNAs, and microRNAs (miRs) could be integrated into EVs, which makes EVs an optimum intrinsic systematic delivery vehicle [7]. Furthermore, a prior study revealed that miR-22-3p was enriched in EVs derived from BMSCs [8]. Furthermore, miR-22-3p has been detected as one of top 20 abundant miRs in plasma-derived EVs [9]. More recently, miR-22-3p is engaged in osteogenic differentiation of adipose-derived stem cells, as detected by the gene microarray in a study [10]. FTO alpha-ketoglutarate dependent dioxygenase (FTO), one of the crucial elements in modulating body weight and fat mass, is correlated with obesity and body mass index (BMI) [11]. FTO is widely known for its function in fat deposition mostly by influencing adipogenesis, and miR-149-3p-targeted downregulation of FTO can lead to the inhibition of osteogenic differentiation [12]. FTO suppression could reportedly give rise to the aggregation of m6A on MYC transcripts, which further reduced stabilization of MYC mRNA and inhibition of MYC pathway [13]. MYC has a fundamental role in nearly all steps involved in the oncogenic processes, covering cell proliferation, cell death, differentiation, and metabolism [14]. As a major oncogenic transcription factor of MYC family, high expression of *c-MYC* could lead to a reduced osteogenic differentiation [15]. In this study, bioinformatics analysis revealed that FTO was a potential target of miR-22-3p. Therefore, a hypothesis was drawn that BMSC-derived EV miR-22-3p was involved in osteogenic differentiation via MYC pathway by targeting FTO. Therefore, the present study was conducted with the main focus placed on the alteration in the expression of miR-22-3p in BMSCs and investigated the function and underlying mechanism of BMSC-derived EV miR-22-3p in osteogenic differentiation via FTO.

## Materials and methods

### Ethical approval

All animal experimental procedures were conducted in accordance with regulations from ethics committee in the First Hospital of Lanzhou University.

### Cell culture

Human BMSCs were purchased from ScienCell and cultured in normal medium (NM) (Cyagen Bioscience, Santa Clara, CA, USA) consisting of basal medium, 10% fetal bovine serum (FBS), 1% penicillin streptomycin, and 1% glutamine. Cells were placed in a 25-cm^2^ flask (Nest, Wuxi, Jiangsu, China), and incubation was carried out in a humidified incubator (Thermo, Austin, TX, USA) at 37 °C and 5% CO_2_.

### Cell transfection

When the confluence of BMSCs reached 50–60%, cells were transfected with small interfering (si)-negative control (NC), si-FTO, mimic-NC, miR-22-3p mimic, inhibitor-NC, and miR-22-3p inhibitor (GenePharma, Suzhou, China). Cells were transfected in the presence of transfection reagents x-treme (F. Hoffmann-La Roche AG, Basel, Switzerland) and Opti Reduced Serum Medium (Invitrogen, Carlsbad, CA, USA). Twenty-four hours after transfection, the cells were obtained for subsequent experiments.

### Osteogenic differentiation

BMSCs were seeded in 6- or 24-well plates (Nest, Wuxi, Jiangsu, China) to induce osteogenesis. When the confluence of BMSCs reached 80–90%, BMSCs were cultured in osteogenic induction medium (OM) which was supplemented with 10% FBS, 1% glutamine, 0.2% ascorbic acid, 1% penicillin streptomycin, 0.01% dexamethasone, and 1% b-glycerophosphate for 14 days.

### Alizarin red S (ARS) staining and quantitative analysis

ARS staining was employed to detect osteogenesis of BMSCs. Briefly, BMSCs were incubated with ARS staining solution (Cyagen Bioscience, USA) for 20–30 min. BMSCs were observed under an optical microscope (Nikon, Tokyo, Japan). The degree of mineralization of BMSCs was determined. BMSCs were incubated with 100 mM cetylpyridinium chloride (Sigma, St Louis, MO, USA) for 1 h after ARS was solubilized. The absorbance of the released ARS was detected at 570 nm using a microplate reader (Tecan, Männedorf, Zürich, Switzerland).

### Alkaline phosphatase (ALP) staining

The medium was removed after BMSCs in a 24-well plate and was washed with phosphate buffer saline (PBS). Cells were then fixed with 95% ethanol and stained with ALP solution, followed by 4-h incubation in a 37 °C incubator. Then, 2% cobalt nitrate (Tianli Chemical Reagents, Tianjin, China) and ammonium sulfide (Tianli Fuyu Fine Chemical, China) were added. BMSCs were observed under an optical microscope (Nikon, Tokyo, Japan). The cells then underwent incubation with 10 mM p-nitrophenyl phosphate (Meilunbio, Dalian, China) for 30 min for quantitative analysis. Finally, the absorbance value (420 nm) was determined by a spectrophotometry reader.

### Reverse transcription quantitative polymerase chain reaction (RT-qPCR)

Total RNA was extracted from the cells using Trizol reagent (Invitrogen, Carlsbad, CA, USA). The quantity and quality of RNAs were assessed with the use of a NanoDrop spectrophotometer (Thermo, Austin, TX, USA). Then, 0.5 mg RNA was reversely transcribed into cDNA using a High Capacity cDNA Reverse Transcription Kit (Applied Biosystems, NY, USA). Target genes were detected by real-time quantitative PCR using SYBR Green Master reagents (F. Hoffmann-La Roche AG, Basel, Switzerland). Glyceraldehyde-3-phosphate dehydrogenase (GAPDH) and U6 were used as the internal controls for examining levels of genes and miR respectively. The relative expression of target genes was evaluated using the 2^△△CT^ method. To detect miR expression in EVs, 20 fmol of synthetic cel-miR-22-3p was added to EVs from an equal number of cells during RNA extraction. The primer sequences are shown in Table 1.

**Table 1:** RT-qPCR primers

Targets	Forward (5′-3′)	Reverse (5′-3′)
miR-22-3p	AAGCTGCCAGTTGAAGAACTGT	/
RUNX2	ACTTCCTGTGCTCGGTGCT	GACGGTTATGGTCAAGGTGAA
OPN	CTCCATTGACTCGAACGACTC	CAGGTCTGCGAAACTTCTTAGAT
OCN	TGAGAGCCCTCACACTCCTC	CGCCTGGGTCTCTTCACTAC
FTO	ACTTGGCTCCCTTATCTGACC	TGTGCAGTGTGAGAAAGGCTT
U6	CGCTTCGGCAGCACATATACTAAAATTGGAAC	/
GAPDH	ATCCCATCACCATCTTCC	GAGTCCTTCCACGATACCA

*miR-22-3p* micro-22-3p, *RUNX2* RUNX family transcription factor 2, *OPN* secreted phosphoprotein 1, *OCN* ocnus, *FTO* FTO alpha-ketoglutarate dependent dioxygenase, *GAPDH* glyceraldehyde-3-phosphate dehydrogenase

### Isolation and purification of EVs

When BMSCs reached 80% confluence, the medium was replaced with EV-free medium, and the supernatant was collected 48 h later. Subsequently, differential centrifugation and filtration were carried out to isolate EVs from supernatant of BMSCs. Cell supernatant was centrifuged at 2000*g* for 20 min and then at 10,000*g* for 40 min and filtered through a 0.22-μm sterilizing filter (Millipore, Billerica, MA, USA). The supernatant was then ultra-centrifuged at 100,000*g* for 70 min, resuspended in PBS, centrifuged at 100,000*g* for 70 min to remove any residual RNA, and diluted with a mixture containing PBS and RNAse I (Invitrogen, Carlsbad, CA, USA). To measure concentration of EVs, EVs were lysed in Radio-Immunoprecipitation Assay lysis buffer and tested using a Pierce bicinchoninic acid Protein Assay Kit (Thermo Fisher Scientific Inc., Waltham, MA, USA).

### Identification of EVs

The morphology of EVs was observed under a transmission electron microscope (TEM). The BMSC-derived EVs were dropped into a carbon-coated copper mesh and air dried. The mixture was subject to negative staining twice with 1% uranyl acetate, and images were obtained with a HT7700 transmission electron microscope (Hitachi, Tokyo, Japan) at 120 kV. The particle size and concentration of EVs were determined by Nanoparticle Tracking Analysis (NTA). Briefly, EVs were measured using the Zetaview system (Particle Metrix GmbH, Microtrac, Meerbusch, Germany), and the results were analyzed using NTA analysis software (Zetaview, version 8.04.02). Immunoblotting was employed to detect EV-specific markers (CD9, CD63, β-tubulin, and histone 1).

### EV uptake analysis

EVs were stained with red fluorescent PKH26. Briefly, 20 μL EVs were isolated from BMSCs and diluted in 1 mL diluent C to 50 or 100 μg/mL, and 4 μL PKH26 dye was diluted in 1 mL diluent C. Both dilutions were gently mixed for 4 min, added with 2 mL of 0.5% bovine serum albumin. The labeled EVs underwent centrifugation at 100,000*g* for 70 min. The EVs were collected after EVs were enriched in a sucrose density range of 1.13–1.19 g/mL. BMSCs were then incubated with EVs for 24 h. Cells were fixed in 4% paraformaldehyde for 10 min, and the nuclei were stained with 4′,6-diamino-2-phenylindole solution at a concentration of 1 μg/mL. Finally, EV uptake was viewed under an LSM5 exciter confocal laser scanning microscope (Carl Zeiss AG, Germany). Quantitative analysis of fluorescence signal was conducted by Image J software.

### Dual-luciferase report gene assay

The 3′-untranslated region (UTR) of FTO containing predicted miR-22-3p binding site was synthesized and cloned into pcDNA3.1 (+) containing the firefly luciferase reporter gene. An FTO wild-type (WT) luciferase reporter plasmid was constructed at the downstream position of the luciferase reporter gene. A site-directed mutagenesis kit (SBS Genetech Co., Ltd., Beijing, China) was used to mutate miR-22-3p binding site in 3′-UTR of FTO [FTO-mutant (MUT) luciferase reporter plasmid]. When HEK-293 T grew to 70%–80% confluence in a 48-well plate, the cells were transfected with 400 ng FTO-MUT or FTO-WT expressing plasmid, 40 ng firefly luciferase reporter plasmid and 4 ng pRL-TK, a plasmid expressing *Renilla* luciferase (Promega, Madison, WI, USA). Luciferase activity was measured 24 h after transfection with the use of a dual-luciferase reporter system. All luciferase values were relative to *Renilla* luciferase values and expressed as fold changes from basal activity.

### Immunoblotting

Proteins from cells or EVs were separated on sulfate polyacrylamide gel electrophoresis gel and transferred to a polyvinylidene fluoride membrane. Thereafter, the membrane was probed with anti-CD9 (# ab92726, abcam, Cambridge, UK), CD63 (# ab134045, abcam), β-tubulin (# SC-5274, Santa Cruz Biotechnology Inc., Santa Cruz, CA, USA), and histone 1 (# SC-8030, Santa Cruz Biotechnology Inc., Santa Cruz, CA, USA), RUNX Family Transcription Factor 2 (RUNX2; # ab23981, 1: 1000, abcam), osteopontin (OPN; # ab8448, 1: 1000, abcam), osteocalcin (OCN; # ab93876, 1: 500, abcam), FTO (# ab94482, 1: 1000, abcam), C-MYC (# ab32072, 1: 1000, abcam), phosphorylated AKT (Ser473) (# 4060; 1: 1000; Cell Signaling, Danvers, MA), Akt (# 4691; 1: 1000; Cell Signaling), phosphorylated PI3K (abs130868; 1: 1000; Absin, Absin Bioscience Inc., Shanghai, China) and PI3K (abs119725; 1: 1000; Absin), and GAPDH (# ab9485, Abcam) were at 4 °C overnight and re-probed with secondary anti-rabbit (# 7074, Cell Signaling) and anti-mouse (# 7076, Cell Signaling) antibodies for 1 h at room temperature. The protein bands were observed using electrogenerated chemiluminescence kit (CoWin BioSciences, Beijing, China).

### Establishment of osteoporotic mice model

A total of 120 C57BL/6 J mice (aged 6–8 weeks, weighing 18–20 g) were randomly divided into the sham operation group (*n* = 12) or ovariectomized (OVX) group (*n* = 108). Mice in the OVX group underwent bilateral ovariectomy, while mice in the sham group underwent adipose tissue resection near the ovary. After 4 weeks, mouse models of osteoporosis were successfully established. Successfully established modeled mice were randomly divided into OVX group, OVX + PBS group, OVX + BMSC-EV group, OVX + BMSC-EV/inhibitor-NC group, OVX + BMSC-EV/miR-22-3p inhibitor group, OVX + miR-22-3p inhibitor + dimethyl sulfoxide (DMSO) group, OVX + miR-22-3p inhibitor + LY294002 group, OVX + BMSC-EV/inhibitor-NC + DMSO group, and OVX + BMSC-EV/inhibitor-NC + LY294002 group (*n* = 12/group). EVs were injected into mice via the femoral bone marrow periosteum [16], and 20 μL EV suspension was injected twice a week. LY294002 was injected intraperitoneally into rats at a dose of 2 ng/mL/kg once a day. After 2 weeks under sterile conditions, the femurs obtained from the mice were washed and the BMSCs were collected for ARS and ALP staining.

### Bioinformatics analysis

The downstream genes targeted by human miRs were predicted with the use of DIANA TOOLS (miTG score > 0.6) (http://diana.imis.athena-innovation.gr/DianaTools), RAID (http://www.rna-society.org/raid2/index.htmL), miRWalk (Bindingp = 1, energy < − 20, accessibility < 0.01, au > 0.55) (http://mirwalk.umm.uni-heidelberg.de/), TargetScan (cumulative weighted context ++ score < 0) (http://www.targetscan.org/vert_71/), RNA22 (https://cm.jefferson.edu/rna22/), and starBase (http://starbase.sysu.edu.cn/). DIANA TOOLS (miTG score > 0.5) and miRWalk (energy < − 25) were employed to predict the downstream genes of mouse miR. Next, genes targeted by both human and mouse miRs were compared to obtain the key downstream genes. The downstream pathways that might be regulated by key genes were screened using methods described in pre-existing literature and verified using MEM (https://biit.cs.ut.ee/mem/index.cgi) and KOBAS (http://kobas.cbi.pku.edu.cn/).

### Statistical analysis

SPSS 21.0 (IBM Corp., Armonk, NY, USA) was utilized for statistical analysis. The measurement data conforming to normal distribution and homogeneous variance were expressed by mean ± standard deviation of three independent tests. Unpaired *t* test was adopted for the analysis of the differences between two experimental groups, while one-way analysis of variance (ANOVA) was utilized to compare data among multiple groups, followed by Tukey’s post hoc test. Repeated measures ANOVA was used to analyze data among multiple groups at different time points, followed by Bonferroni posttest. *p* < 0.05 was considered as a statistically significant value.

## Results

### Elevation of miR-22-3p promotes osteogenic differentiation of BMSCs

**Fig. 1:**
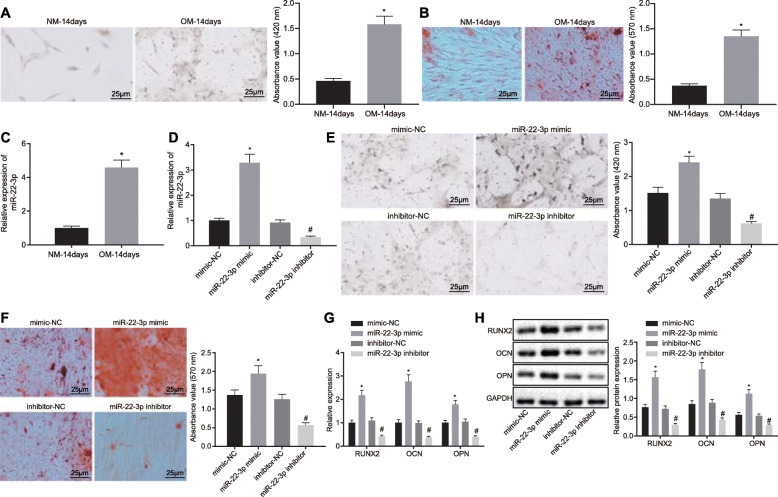
Elevation of miR-22-3p enhances osteogenic differentiation of BMSCs. **a** BMSCs were cultured in NM and OM for 14 days for ALP staining (× 400). **b** BMSCs were cultured in NM and OM for 14 days for ARS staining (× 200). **c** BMSCs were cultured in NM and OM for 14 days, and RT-qPCR was used to detect miR-22-3p expression. **d** Transfection efficiency examination was determined using RT-qPCR in the mimic-NC miR-22-3p mimic, inhibitor-NC, or miR-22-3p inhibitor groups. **e** BMSCs were stained by ALP (× 400). **f** BMSCs was stained by ARS staining in the mimic-NC miR-22-3p mimic, inhibitor-NC, or miR-22-3p inhibitor groups (× 200). **g** RT-qPCR analysis of RUNX2, OCN, and OPN mRNA expression in BMSCs in the mimic-NC miR-22-3p mimic, inhibitor-NC, or miR-22-3p inhibitor groups. **h** Immunoblotting was adopted to determine RUNX2, OCN, and OPN protein expression in BMSCs in the mimic-NC miR-22-3p mimic, inhibitor-NC, or miR-22-3p inhibitor groups. **p < 0.001 vs. the NM-14 days group; *p < 0.05 vs. the mimic NC group; ^#^*p* < 0.05 vs. the inhibitor-NC group. Comparison between two groups was performed by unpaired *t* test

To investigate whether miR-22-3p was involved in osteogenic differentiation, BMSCs were cultured in NM and OM for 14 days. ALP and ARS staining showed that OM markedly promoted osteoblast differentiation (Fig. 1a, b). RT-qPCR results displayed that OM remarkably upregulated the expression of miR-22-3p (Fig. 1c). To further examine the physiological functions of miR-22-3p in osteogenic differentiation, miR-22-3p was overexpressed and inhibited in BMSCs and was determined using RT-qPCR (Fig. 1d). ALP and ARS analysis of transfected BMSCs (Fig. 1e, f) demonstrated that miR-22-3p elevation led to an increase in ALP activity and matrix mineralization of BMSCs, while the opposite trend was observed in response to miR-22-3p inhibitor. RT-qPCR and immunoblotting results (Fig. 1g, h) also displayed that miR-22-3p mimic upregulated RUNX2 expression in BMSCs (determiner of pluripotent cell differentiation into osteoblasts), OCN (a non-collagenous bone matrix protein that plays an important role in bone metabolism including bone reconstruction and bone mineralization synthesized by osteoblasts), and OPN (a phosphorylated glycoprotein present in the bone matrix, participating in a variety of biological functions such as mineralization, absorption, and formation of bones). However, RUNX2, OCN, and OPN expressions were all reduced in BMSCs in response to miR-22-3p inhibitor. The above results indicated that overexpression of miR-22-3p promoted osteogenic differentiation of BMSCs.

### Optimization for BMSC treatment with EVs at suitable concentration

**Fig. 2:**
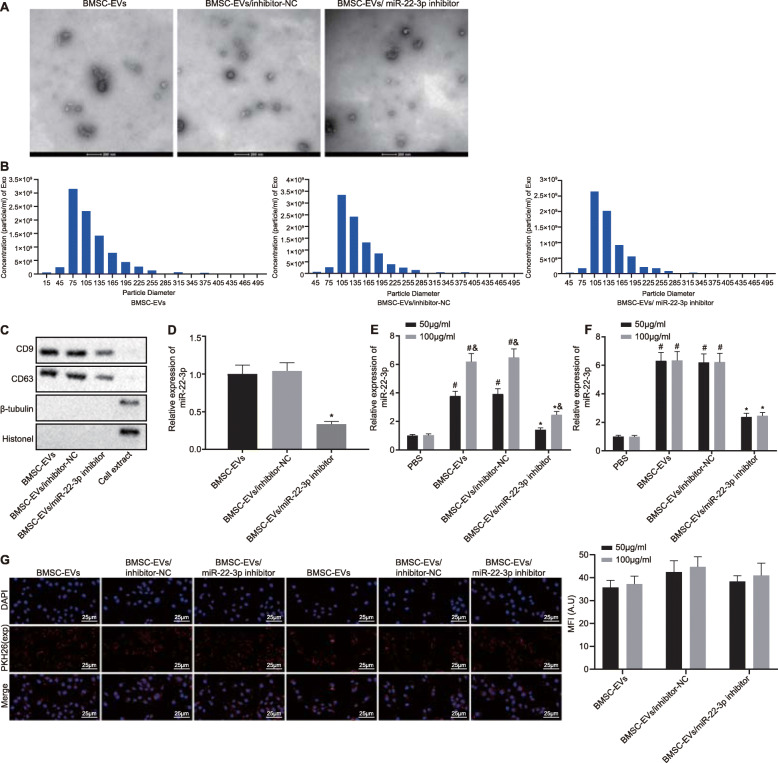
Optimization for BMSC treatment with EVs at 50 μg/mL concentration. BMSC were divided into BMSC-EV/miR-22-3p inhibitor, BMSC-EV/inhibitor-NC group, or BMSC-EV groups. **a** TEM analysis of EVs shows spherical morphology (scale bar = 200 nm). **b** NTA analysis shows the size of EVs. **c** Immunoblotting was adopted to confirm the expression of EV marker protein CD9, CD63, β-tubulin (cytoplasmic marker), or histone 1 (nuclear marker). **d** RT-qPCR was adopted to determined miR-22-3p content in isolated EVs. **e** The content of miR-22-3p in BMSCs was analyzed by RT-qPCR after isolated EVs were incubated with BMSCs for 4 h. **f** The contents of miR-22-3p in BMSCs of 50 μg/mL group and 100 μg/mL group were analyzed by RT-qPCR. **g** Fluorescence microscopy showed that PKH26-labeled EVs (red) were gradually internalized by BMSCs (× 400). *p < 0.05 vs. the BMSC-EV/inhibitor-NC group, ^#^*p* < 0.05 vs. the PBS group, and *p* < 0.05 vs. EVs at the concentration of 100 μg/mL. Comparison between two groups was performed by unpaired *t* test. Comparison of data between multiple groups was performed by one-way ANOVA. Tukey’s was used for post hoc tests

To confirm whether EV-delivered miR-22-3p exerted function on osteogenic differentiation, the expression of miR-22-3p was inhibited in BMSCs, followed by isolation of EVs from BMSCs. TEM and NTA analysis showed that EVs in the BMSC-EV group, BMSC-EV/inhibitor-NC group, and BMSC-EV/miR-22-3p inhibitor group, all exhibited spherical morphology with the size of approximately 75 nm (Fig. 2a, b). In addition, immunoblotting confirmed that EV marker proteins CD9 and CD63 were expressed in EVs, while β-tubulin (cytoplasmic marker) or histone 1 (nuclear marker) expression was hardly detected in EVs (Fig. 2c).

RT-qPCR results displayed that the expression of miR-22-3p in the BMSC-EV/miR-22-3p inhibitor group was markedly lower than that of BMSC-EV/inhibitor-NC group (Fig. 2d). Subsequently, the isolated EVs underwent incubation with BMSCs for 4 h. RT-qPCR results showed that BMSC-EV and BMSC-EV/inhibitor-NC treatment could increase miR-22-3p expression in BMSCs in a dose-dependent manner (Fig. 2e). When the incubation time extended to 24 h, there was no significant difference between the 50 μg/mL-EV group and the 100 μg/mL-EV group (Fig. 2f). Similarly, fluorescence microscopy showed that PKH26-labeled EVs (red) were gradually internalized by BMSCs over time. No marked difference was observed regarding the number of red dots between the 50 μg/mL-EV group and the 100 μg/mL-EV group (Fig. 2g). Therefore, EVs at 50 μg/mL concentration with 24-h incubation were selected for subsequent analysis.

### EV-delivered miR-22-3p inhibitor from BMSCs inhibits osteogenic differentiation

**Fig. 3:**
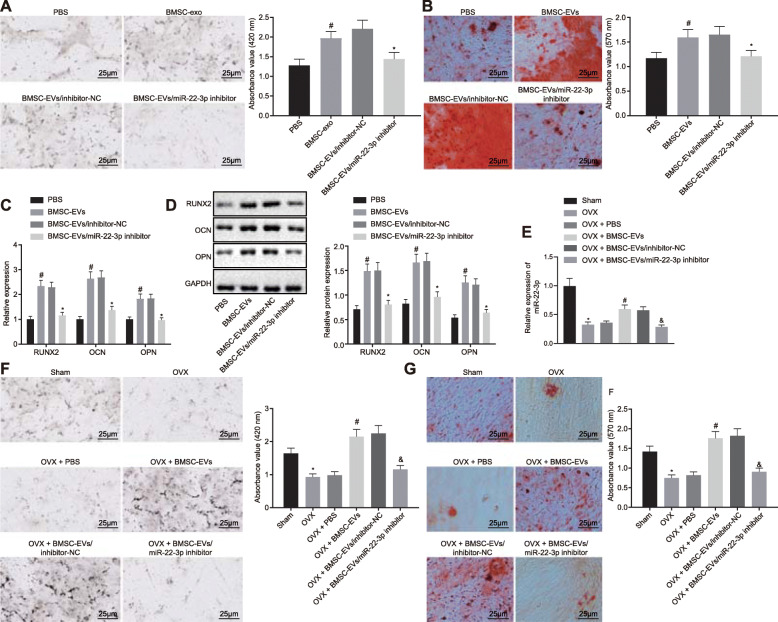
Downregulation of EV-encapsulated miR-22-3p impairs BMSC osteogenic differentiation. **a** The ALP activity of BMSCs were determined using ALP staining in the PBS, BMSC-EV, BMSC-EV/inhibitor-NC, or BMSC-EV/miR-22-3p inhibitor groups (× 400). **b** The extracellular matrix mineralization was determined using ARS staining in the PBS, BMSC-EV, BMSC-EV/inhibitor-NC, or BMSC-EV/miR-22-3p inhibitor groups (× 200). **c**, **d** RT-qPCR and Immunoblotting were adopted to determine the mRNA and protein level of RUNX2, OCN, and OPN in the PBS, BMSC-EV, BMSC-EV/inhibitor-NC, or BMSC-EV/miR-22-3p inhibitor groups. **e** miR-22-3p expression in BMSCs of the sham, OVX + PBS, OVX + BMSC-EV, OVX + BMSC-EV/inhibitor-NC, or OVX + BMSC-EV/miR-22-3p inhibitor groups. **f** ALP activity of BMSCs in mice was determined using ALP staining in the sham, OVX + PBS, OVX + BMSC-EV, OVX + BMSC-EV/inhibitor-NC, or OVX + BMSC-EV/miR-22-3p inhibitor groups (*n* = 12/group) (× 400). **g** ARS staining was adopted to determine the extracellular matrix mineralization of BMSC in mice of the sham, OVX + PBS, OVX + BMSC-EV, OVX + BMSC-EV/inhibitor-NC, or OVX + BMSC-EV/miR-22-3p inhibitor groups (× 200) (*p < 0.05 vs. the sham group or the BMSC-EV/inhibitor-NC group, ^#^*p* < 0.05 vs. the PBS group or the OVX + PBS group, and *p* < 0.05 vs. the OVX + BMSC-EV/inhibitor-NC group). Comparison of data between multiple groups was performed by one-way ANOVA. Tukey’s was used for post hoc tests

The isolated EVs were incubated with BMSCs for 24 h. ALP and ARS staining results showed that compared with the PBS group, the BMSC-EV group had enhanced ALP activity and extracellular matrix mineralization. Compared with the BMSC-EV/inhibitor-NC group, ALP activity and extracellular matrix mineralization of the BMSC-EV/miR-22-3p inhibitor group markedly reduced (Fig. 3a, b). RT-qPCR and immunoblotting showed a significant increase in levels of RUNX2, OCN, and OPN in the BMSC-EV group in comparison with the PBS group. However, compared with the BMSC-EV/inhibitor-NC group, levels of RUNX2, OCN, and OPN were decreased in the BMSC-EV/miR-22-3p inhibitor group (Fig. 3c, d).

To explore the biological role of EVs containing miR-22-3p in bone formation, OVX-induced osteoporotic mouse models were established, with PBS injected as control. To confirm the content of miR-22-3p in BMSCs isolated from model mice, RT-qPCR analysis showed that the expression of miR-22-3p in BMSCs of OVX-induced osteoporotic mice was much lower than that in the sham-operated mice. BMSC-EVs could reverse the decreased expression of miR-22-3p in OVX mice, while the BMSC-EV/miR-22-3p inhibitor possessed weak reversal effects (Fig. 3e). ARS and ALP staining showed that the osteogenic potential of BMSCs of osteoporotic mice was much lower than that of the sham-operated mice. ALP and ARS staining results confirmed that BMSC-EV treatment reversed the reduction of osteoblasts in osteoporotic mice, while the number of osteoblasts reduced in the OVX + BMSC-EV/miR-22-3p inhibitor group (Fig. 3f, g).

### miR-22-3p negatively targets FTO

**Fig. 4:**
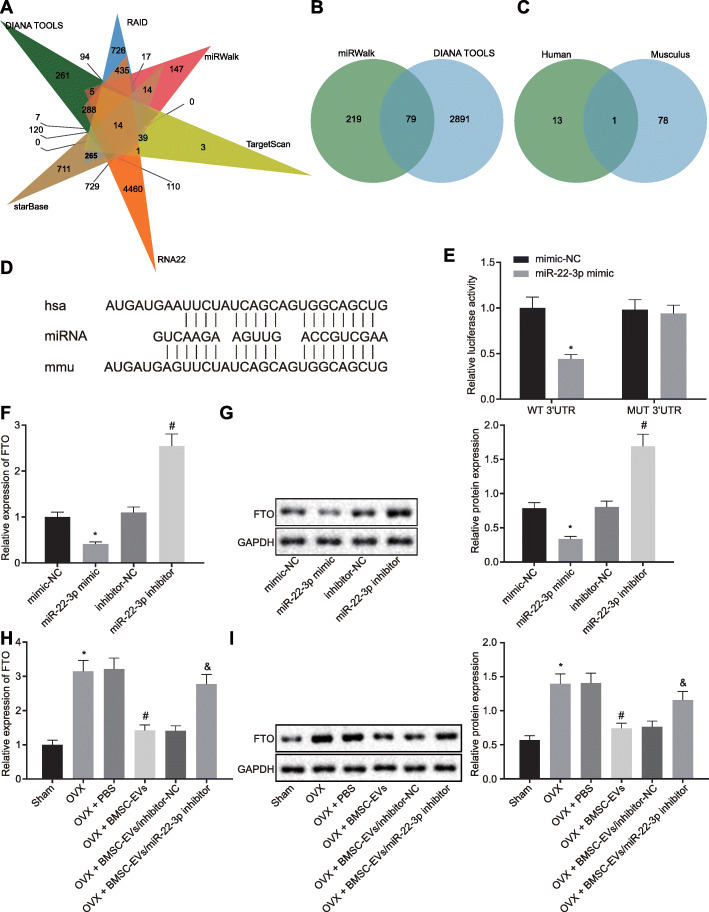
miR-22-3p inversely targets FTO. **a** Venn plot of the downstream target genes of human miR-22-3p predicted by database DIANA TOOLS, RAID, miRWalk, TargetScan, RNA22, and starBase. **b** Venn plot of the downstream target genes of mouse miR-22-3p predicted by database miRWalk and DIANA TOOLS. **c** Venn plot of the downstream genes of both human and mouse miR-22-3p, and the intersection gene is FTO. **d** DIANA TOOLS website analysis to predict the binding site of the human (top) and mouse (bottom) gene FTO to the 3′UTR region of miR-22-3p (middle). **e** Analysis of dual-luciferase reporter assay in HEK-293 T cells. **f**, **g** The mRNA and protein expression of FTP were analyzed by RT-qPCR and immunoblotting. **h**, **i** RT-qPCR and immunoblotting were used to analyze mRNA and protein expression of FTO in mice femoral tissue in each group (*n* = 12/group) (*p < 0.05 vs. the sham group or the BMSC-EV/inhibitor-NC group or the mimic-NC group, ^#^*p* < 0.05 vs. the inhibitor-NC group or the OVX + BMSC-EV/inhibitor-NC group). Comparison between two groups was performed by unpaired *t* test. Comparison of data between multiple groups was performed by one-way ANOVA, and Tukey’s was used for post hoc tests

By using the databases of DIANA TOOLS, RAID, miRWalk, TargetScan, RNA22, and starBase, the downstream target genes of human miR-22-3p were predicted. The 1932, 2911, 430, 591, 7220, and 1365 downstream genes were identified respectively, with 14 genes identified at the intersection (Fig. 4a). Moreover, miRWalk and DIANA TOOLS predicted 296 and 2970 downstream genes of mouse miR-22-3p, respectively. Venn plots were taken to obtain the intersections (79 genes) (Fig. 4b). By comparing the downstream genes of both human and mouse miR-22-3p, FTO was identified as the only gene with a critical expression in both origins (Fig. 4c), and a prior report demonstrated that FTO could inhibit osteogenic differentiation [12]. By using the DIANA TOOLS website, the prediction identified a binding site between miR-22-3p and the 3′-UTR region of FTO in both human and mice (Fig. 4d), which was verified using dual-luciferase reporter gene assay. As predicted, compared with the mimic-NC + WT-3′UTR group, HEK-293 T cells co-transfected with the miR-22-3p mimic and a plasmid containing FTO WT 3′-UTR region presented with a remarkably reduced luciferase activity, while HEK293T cells co-transfected with miR-22-3p mimic and a plasmid containing the 3′-UTR region of FTO MUT showed no significant difference in luciferase activity (Fig. 4e). RT-qPCR and immunoblotting (Fig. 4f, g) showed that miR-22-3p mimic could inhibit FTO expression in BMSCs, but this trend was on the contrary upon treatment with miR-22-3p inhibitor. RT-qPCR and immunoblotting results showed that compared with the sham group, both mRNA and protein expression of FTO in the OVX group showed a potent rise. Compared with the OVX + PBS group, expression of FTO in the OVX + BMSC-EV group was downregulated, while the opposite trend was observed in the OVX + BMSC-EV/miR-22-3p inhibitor group when compared with the OVX + BMSC-EV/inhibitor-NC group (Fig. 4h, i).

### FTO silencing can rescue the suppressed osteogenic differentiation by miR-22-3p inhibitor

**Fig. 5:**
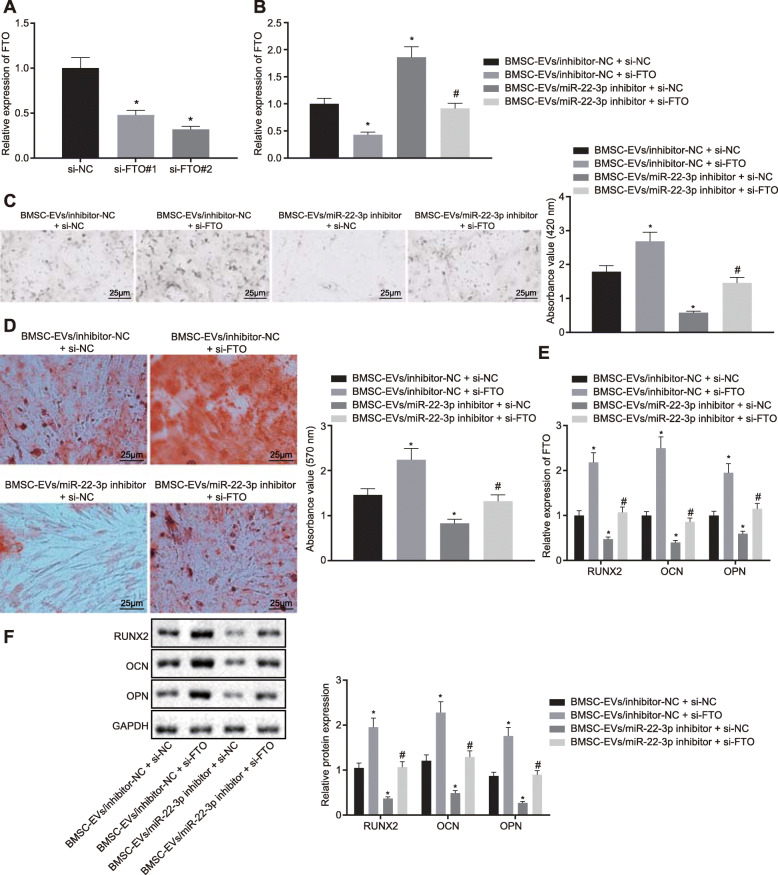
FTO repression rescues the promotion of osteogenic differentiation by miR-22-3p. BMSC were divided into the BMSC-EV/inhibitor-NC + si-NC, BMSC-EV/inhibitor-NC + si-FTO, BMSC-EV/miR-22-3p inhibitor + si-NC group, and BMSC-EV/miR-22-3p inhibitor + si-FTO groups. **a** RT-qPCR was adopted to determine the knockdown efficiency of the si-FTO # 1 and si-FTO # 2 groups. **b** Detection of transfection efficiency by RT-qPCR. **c** ALP activity of BMSCs was determined using ALP staining (× 400). **d** ARS staining was adopted to determine the extracellular matrix mineralization of BMSC (× 200). **e** RT-qPCR was used to detect the mRNA level of RUNX2, OCN, and OPN. **f** Immunoblotting was adopted to determine the protein level of RUNX2, OCN, and OPN (*p < 0.05 vs. the BMSC-EV/inhibitor-NC + si-NC group, ^#^*p* < 0.05 vs. the BMSC-EV/miR-22-3p inhibitor + si-NC group). Comparison between multiple groups was performed by one-way ANOVA, and Tukey’s was used for post hoc tests

FTO silencing was carried out following the incubation of the BMSCs with EVs. Firstly, two si-FTOs were designed and the silencing effect of si-FTO # 2 was found to be more significant (Fig. 5a) and was therefore selected for subsequent experiments. RT-qPCR was used to examine the transfection efficiency, with the results illustrated in Fig. 5b. Compared with the BMSC-EV/inhibitor-NC + si-NC group, the expression of FTO in the BMSC-EV/inhibitor-NC + si-FTO group showed a marked decline. However, the expression of FTO in the BMSC-EV/miR-22-3p inhibitor + si-NC group was increased. Compared with the BMSC-EV/miR-22-3p inhibitor + si-NC group, the expression of FTO in the BMSC-EV/miR-22-3p inhibitor + si-FTO group was reduced. ALP and ARS staining (Fig. 5c, d) were then performed, and the results showed that compared with the BMSC-EV/inhibitor-NC + si-NC group, the ALP staining and activity and extracellular matrix mineralization were enhanced in the BMSC-EV/inhibitor-NC + si-FTO group and decreased in the BMSC-EV/miR-22-3p inhibitor + si-NC group. However, the opposite trend was observed in the BMSC-EV/miR-22-3p inhibitor + si-FTO group compared with the BMSC-EV/miR-22-3p inhibitor + si-NC group. The results from RT-qPCR and immunoblotting (Fig. 5e, f) showed that compared with the BMSC-EV/inhibitor-NC + si-NC group, the mRNA and protein expression of RUNX2, OCN, and OPN in the BMSC-EV/inhibitor-NC + si-FTO group increased, while the expression of RUNX2, OCN, and OPN in the BMSC-EV/miR-22-3p inhibitor + si-NC group decreased significantly. The BMSC-EV/miR-22-3p inhibitor + si-FTO group presented with the opposite trend compared with the BMSC-EV/miR-22-3p inhibitor + si-NC group. These results suggested that the silencing of FTO could rescue the suppression of osteogenic differentiation by miR-22-3p inhibitor-modified EVs.

### Elevation of miR-22-3p inhibits MYC/PI3K/AKT pathway by targeting FTO

**Fig. 6:**
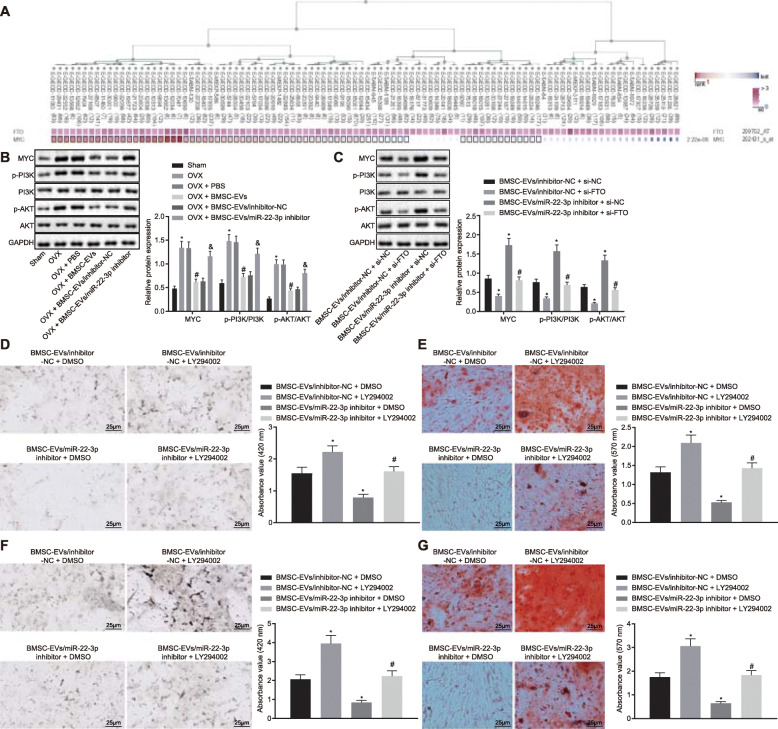
miR-22-3p inactivates the MYC/PI3K/AKT pathway by targeting FTO. **a** The co-expression pattern between FTO and MYC was generated by the prediction of MEM website (*p* = 2.22E−06). **b** Immunoblotting was adopted to determine the protein expression of MYC, phosphorylated PI3K/PI3K, and phosphorylated AKT/AKT in mice femur in the OVX + PBS, OVX + BMSC-EV, OVX + BMSC-EV/inhibitor-NC, or BMSC-EV/miR-22-3p inhibitor groups (*n* = 12/group). **c** Immunoblotting of the protein expression of MYC, phosphorylated PI3K/PI3K, and phosphorylated AKT/AKT in BMSC-EV/inhibitor-NC + si-NC, BMSC-EV/inhibitor-NC + si-FTO, BMSC-EV/miR-22-3p inhibitor + si-NC, or BMSC-EV/miR-22-3p inhibitor + si-FTO groups. **d** ALP staining in BMSC-EV/inhibitor-NC + si-NC, BMSC-EV/inhibitor-NC + si-FTO, BMSC-EV/miR-22-3p inhibitor + si-NC, or BMSC-EV/miR-22-3p inhibitor + si-FTO groups (× 400). **e** ARS staining was adopted to determine the extracellular matrix mineralization of BMSC (× 200). **f** ALP staining of BMSCs in the OVX-BMSC-EV/inhibitor-NC + DMSO, OVX-BMSC-EV/inhibitor-NC + LY294002, OVX-BMSC-EV/miR-22-3p inhibitor + DMSO, or OVX-BMSC-EV/miR-22-3p inhibitor + LY294002 groups (*n* = 12/group) (× 400). **g** ARS staining of BMSCs in the OVX-BMSC-EV/inhibitor-NC + DMSO, OVX-BMSC-EV/inhibitor-NC + LY294002, OVX-BMSC-EV/miR-22-3p inhibitor + DMSO, or OVX-BMSC-EV/miR-22-3p inhibitor + LY294002 groups (*n* = 12/group) (× 200) (*p < 0.05 vs. the BMSC-EV/inhibitor-NC + DMSO group, ^#^*p* < 0.05 vs. the BMSC-EV/miR-22-3p inhibitor + DMSO group). The comparison between multiple groups was performed by one-way ANOVA, and Tukey’s was used for post hoc tests. LY294002 (20 μM), a PI3K/AKT pathway inhibitor, was prepared by Sigma-Aldrich (Shanghai, China)

It has been reported that FTO is capable of removing the m6A modification at the 5′ end of MYC gene, stabilize the mRNA of MYC, and promote MYC expression [13], and MYC can activate PI3K/AKT pathway in normal cells [17]. To investigate whether miR-22-3p inhibited MYC/PI3K/AKT pathway by targeting FTO, we initially found significant co-expression pattern between FTO and MYC based on the findings obtained from MEM analysis (*p* = 2.22E−06) (Fig. 6a), after which KOBAS was used to perform MYC enrichment analysis, and it was confirmed that MYC was enriched in PI3K/AKT pathway (*p* = 8.63E−03). Then, the activation of MYC/PI3K/AKT pathway was tested in mouse femur from each group. As shown in Fig. 6b, compared with the sham group, the expression of MYC, phosphorylated PI3K/PI3K, and phosphorylated AKT/AKT proteins in the OVX group increased. However, compared with the OVX + PBS group, MYC, phosphorylated PI3K/PI3K, and phosphorylated AKT/AKT protein expressions in the OVX + BMSC-EV group was downregulated. Compared with the OVX + BMSC-EV/inhibitor-NC group, MYC, phosphorylated PI3K/PI3K, and phosphorylated AKT/AKT protein expressions in the OVX + BMSC-EV/miR-22-3p inhibitor group was upregulated. As shown in Fig. 6c, compared with the BMSC-EV/inhibitor-NC + si-NC group, the protein expression of MYC, phosphorylated PI3K/PI3K, and phosphorylated AKT/AKT in the BMSC-EV/inhibitor-NC + si-FTO group had markedly decreased, while MYC, phosphorylated PI3K/PI3K, and phosphorylated AKT/AKT expressions in the BMSC-EV/miR-22-3p inhibitor + si-NC group was elevated. Compared with the BMSC-EV/miR-22-3p inhibitor + si-NC group, MYC, phosphorylated PI3K/PI3K, and phosphorylated AKT/AKT expressions in the BMSC-EV/miR-22-3p inhibitor + si-FTO group reduced. The aforementioned findings highly indicated that miR-22-3p targeted FTO, thereby inhibiting the MYC/PI3K/AKT pathway. In addition, the effect of miR-22-3p on osteogenic differentiation of BMSCs through FTO/MYC/PI3K/AKT pathway was further analyzed. BMSCs were treated with PI3K/AKT inhibitor after incubation with EVs. The results of ALP and ARS staining are shown in Fig. 6d, e. Compared with the BMSC-EV/inhibitor-NC + DMSO group, ALP staining and activity and extracellular matrix mineralization in the BMSC-EV/inhibitor-NC + LY294002 group were enhanced. However, ALP staining and activity and extracellular matrix mineralization in the BMSC-EV/miR-22-3p inhibitor + DMSO group were reduced, while the BMSC-EV/miR-22-3p inhibitor + LY294002 group was observed to have the opposite trend compared with the BMSC-EV/miR-22-3p inhibitor + DMSO group. In vivo experiments also showed upregulated miR-22-3p from BMSC-derived EVs targeted FTO, thereby inhibiting MYC/PI3K/AKT pathway and promoting osteogenic differentiation (Fig. 6f, g).

## Discussion

The differentiation of BMSCs is involved in osteogenesis and is an important factor involved in normal growth and wound healing of the bone tissue, and the loss of BMSC function may lead to the delay of fracture healing and further enhance pathological changes of the bone tissue [18]. EVs have been recognized for its role in cell-to-cell signal transductions, organ interlinkages, and tissue homeostasis, which is accomplished by the interchanging of molecules among cells, such as proteins, lipids, and miRs [19]. A previous study identified that there exists a correlation between MSC-derived EVs containing miRs and osteogenic differentiation [20]. Therefore, this study aimed to explore the effect of BMSC-derived EV containing miR-22-3p on osteogenic differentiation. Consequently, it was elucidated in our study that EV-encapsulated miR-22-3p from BMSCs resulted in the inactivation of MYC/PI3K/AKT pathway via FTO inhibition, promoting osteogenic differentiation.

Initially, we found that elevated miR-22-3p could promote osteogenic differentiation, evidenced by upregulated RUNX2, OCN, and OPN levels as well as increased ALP activity and matrix mineralization. Quantification of osteogenic differentiation of BMSCs is conducted by examining ALP activities, matrix mineralization, and cell numbers [21]. ARS staining is specifically utilized to characterize calcium mineralization [22]. RUNX2 belongs to the RUNX family and plays an important role in osteoblast differentiation and skeletal formation [23]. Moreover, RUNX serves as a biomarker for early stage of osteogenic differentiation while OCN and OPN are both osteoblast markers [24]. Therefore, induction of osteogenic differentiation would be accompanied by upregulation of RUNX1, OCN, and OPN. A previous study has highlighted the presence of a strong association between miR-22-3p and osteogenesis of adipose-derived stem cells [10]. Another important finding revealed that patients suffering from fractures have low expression of miR-22-3p with inhibited osteogenic differentiation, suggesting that miR-22-3p promotes osteogenic differentiation [25].

In addition, we also found that miR-22-3p could adversely target FTO, inactivating the MYC /PI3K/AKT pathway, thereby promoting osteogenic differentiation. The involvement of FTO in fat aggregation is mainly by modulating adipogenesis, and suppression or mutation of FTO could lead to a decrease in body weight and fat aggregation [12]. FTO expression is commonly upregulated in bone marrows during the period of aging and osteoporosis [26]. Furthermore, FTO inhibition caused by miR-149-3p promoted osteogenic differentiation of BMSCs [12]. A recent study has reported FTO knockdown could result in the suppression of MYC expression, while ectopic expression of WT FTO could induce MYC expression [13]. A series of studies have implicated that MYC particularly interacts with PI3K activation in the presence of abnormal cell proliferation and transformation [27]. In healthy cells, MYC can activate PI3K/AKT pathway [17], whereas accumulating evidence has demonstrated that inactivated PI3K/AKT pathway is related to diverse biological events, such as high-glucose increased metastasis, diabetes, and bone differentiation [28–30]. Moreover, a prior study suggested that PI3K/AKT pathway was associated with downregulated osteogenic differentiation [31].

The last important finding from our study showed that EV-delivered miR-22-3p from BMSCs promoted osteogenic differentiation. It has been elaborated that BMSC-derived EVs modulated osteogenic differentiation in vitro and induced bone regeneration in vivo [19]. Similarly, another study also detected the osteogenic effect of EVs derived from BMSCs on steroid-induced osteonecrosis of the femoral head [5]. Moreover, MSC-derived EVs containing miRs were reported to mediate osteogenic differentiation [20]. For example, EVs derived from miR-375-overexpressing human adipose mesenchymal stem cells could promote bone regeneration [32]. Moreover, EVs derived from BMSCs were observed to have an abundant expression of miR-22-3p [8]. These finding suggested that EV derived from miR-22-3p-overexpressing BMSCs promoted osteogenic differentiation.

## Conclusions

**Fig. 7:**
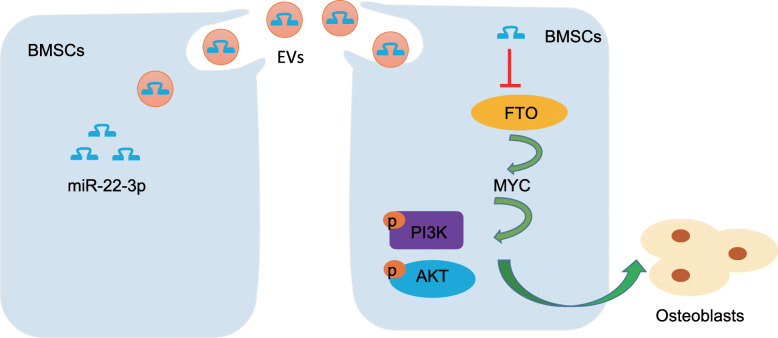
Mechanism, miR-22-3p encapsulated in BMSCs-derived EVs inhibits MYC/PI3K/AKT pathway and promotes osteogenic differentiation by targeting FTO

In summary, we found that miR-22-3p in BMSC-derived EVs can suppress MYC/PI3K/AKT pathway to stimulate osteogenic differentiation by targeting FTO (Fig. 7). However, miRs from EVs present with a complicated readout in both physiological and pathophysiological status within a living organism. A solid conclusion of detected alterations in miRs is commonly challenging, since most of EV-derived miRs are universally expressed, making it difficult to trace the specific tissue. Therefore, tissue-specific miRs should be considered in upcoming studies to provide a clear demonstration of the specific roles of certain miRs.

## Data Availability

The primary data for this study are available from the authors on direct request.

## Abbreviations

BMSCs: Bone mesenchymal stem cells
miR: microRNA
EVs: Extracellular vesicle
FTO: FTO alpha-ketoglutarate dependent dioxygenase
BMI: Body mass index
NM: Normal medium
ARS: Alizarin red S
ALP: Alkaline phosphatase
TEM: Transmission electron microscope
NTA: Nanoparticle Tracking Analysis
MUT: Mutant
WT: Wild-type
ECL: Chemiluminescence
ANOVA: Analysis of variance
